# Developing a large‐scale quality improvement program for thyroid cancer surgery

**DOI:** 10.1002/wjs.12367

**Published:** 2024-10-15

**Authors:** Catherine B. Jensen, Elizabeth M. Bacon, Lauren N. Krumeich, Hunter J. Underwood, David T. Hughes, Paul G. Gauger, Richard Burney, Susan C. Pitt

**Affiliations:** ^1^ Department of Surgery University of Michigan Ann Arbor Michigan USA; ^2^ Center for Health Outcomes and Policy University of Michigan Ann Arbor Michigan USA; ^3^ National Clinician Scholars Program University of Michigan Ann Arbor Michigan USA; ^4^ Department of Surgery University of Wisconsin‐Madison Madison Wisconsin USA; ^5^ Michigan Surgical Quality Collaborative Ann Arbor Michigan USA

**Keywords:** collaborative, quality improvement, quality metrics, thyroid cancer

## Abstract

**Background:**

Surgical quality improvement (QI) plays a critical role in optimizing patient outcomes and reducing healthcare costs. QI programs focusing specifically on thyroid cancer surgical care are lacking. This study aimed to (a) select and introduce surgical quality indicators for thyroid cancer and (b) identify areas for QI at the state‐level.

**Methods:**

A multidisciplinary team of thyroid cancer and QI experts selected 10 thyroid cancer‐specific quality indicators and assessed the quality of thyroid cancer surgical care compared to current national guidelines. Analysis of the first year (January–December 2023) of data collection was performed using descriptive statistics.

**Results:**

The thyroid cancer quality indicators included preoperative cytology, postoperative pathology, staging, cancer size, margin status, extrathyroidal extension, lymph nodes, postoperative complications within 30 days, documented follow‐up treatment, and documented surveillance plans. 112 surgeons performed 360 thyroidectomies for thyroid cancer at 51 hospitals. Preoperative cytology was not performed in 34.3% (*n* = 103) of cases with thyroid cancer on final pathology. When the extent of surgery was evaluated by papillary cancer size, 50.0% (*n* = 38) of patients with <1 cm cancers underwent total thyroidectomy, and 13.8% (*n* = 4) with >4 cm underwent thyroid lobectomy. Positive margins were found in 16.2% (*n* = 53). Postoperatively, 19.2% (*n* = 69) of patients lacked documented follow‐up, and 18.6% (*n* = 67) lacked thyroid cancer surveillance plans.

**Conclusions:**

Establishing a dedicated QI program for thyroid cancer provides a previously unharnessed opportunity to enhance the quality of thyroid cancer surgical care. Statewide surgical quality collaboratives offer a model for establishing thyroid cancer QI initiatives across diverse healthcare settings in other states and countries.

## 1 INTRODUCTION

Surgical quality improvement (QI) plays a critical role in optimizing patient outcomes and reducing healthcare costs.^1^ Unlike surgical outcomes, which focus on specific endpoints, surgical quality encompasses a broader range of indicators, including process and structural measures that contribute to the overall quality of care.^2^, ^3^ QI initiatives implemented as part of surgical quality collaboratives (SQCs) have demonstrated utility in improving cancer care.^4^, ^5^, ^6^, ^7^, ^8^, ^9^ One example is the application of enhanced recovery after surgery (ERAS) pathways with subsequent decreased length of stay for patients undergoing colectomy for colon cancer.^8^, ^9^ Surgeon‐directed interventions employed through SQCs have also decreased re‐excision rates post‐mastectomy in breast cancer and reduced the utilization of low value imaging in men with metastatic prostate cancer.^5^, ^10^

Compared to these other malignancies, thyroid cancer presents unique challenges in assessing surgical quality due to its indolent nature and high survivability.^11^ Over the past 2 decades, changes in thyroid cancer management have aimed to address concerns about overdiagnosis and overtreatment of small, low‐risk malignancies.^12^ Current national quality registries are largely comprised of academic centers and lack indicators critical to measuring and improving the quality of surgery for thyroid cancer.^13^ To the best of our knowledge, no dedicated QI programs exist specifically focusing on improving surgical care of thyroid cancer. A collaborative QI program focused on surgery for thyroid cancer could provide a unique opportunity to improve the quality of thyroid cancer surgical care by encouraging adherence to evidence‐based guidelines and potentially improving clinical outcomes.

To address this gap and identify targets for QI, we aimed to leverage an established statewide SQC to develop and implement a thyroid cancer‐specific surgical quality program. The objectives of this study were to (a) select and implement a collection of thyroid cancer‐specific surgical quality indicators, (b) report on 1 year of data collection establishing proof of concept and (c) identify priorities for future QI initiatives.

## 2 MATERIALS AND METHODS

### 2.1 Setting

The Michigan surgical quality collaborative (MSQC) is a statewide QI program funded by blue cross and blue shield of Michigan (BCBSM) focused on improving surgical outcomes and healthcare utilization.^14^ The MSQC consists of 68 participating hospitals across the state, comprising a diverse mix of healthcare settings including academic, community, urban, and rural hospitals. Each participating hospital receives funding from BCBSM to support a trained nurse abstractor to collect patient data, perioperative processes of care, and 30 day outcomes. Surgical procedures are selected by a stratified sampling method to capture a representative portion of cases. Sampling of thyroid operations followed the existing MSQC case abstraction model. Smaller sites abstract 10% of their thyroid cases because of the limited eligible case volume, while larger volume sites abstract a smaller percentage of thyroidectomy cases. Data are abstracted more than 30 days postoperatively in a standardized fashion across the collaborative and available in real time for utilization in benchmarking and QI initiatives. The MSQC has a proven track record of leveraging these data to improve surgical quality and patient outcomes across various surgical specialties.^15^, ^16^, ^17^

Prior to January 2023, data collection for patients undergoing thyroidectomy was limited to length of stay, surgical site infection, emergency department visit, readmission, reoperation, and mortality. While these variables provide valuable information, they did not capture nuances specific to thyroid cancer care, such as the appropriateness of the surgical procedure or plans for cancer surveillance. Recognizing this gap, the MSQC sought to expand its data collection to include thyroid cancer‐specific quality indicators.

### 2.2 Identification of quality indicators

To identify thyroid cancer‐specific quality indicators, we performed a comprehensive literature review and compiled a list of potential variables spanning the continuum of thyroid cancer surgical care, including preoperative evaluation, operative approach, pathologic assessment, and postoperative management.^11^, ^18^, ^19^, ^20^ The initial list of candidate variables included the performance of preoperative fine‐needle aspiration (FNA) biopsy, lymph node number, margin status, and use of postoperative radioactive iodine therapy.

To refine the list of quality indicators and ensure their relevance and feasibility for implementation with the MSQC, we convened a multidisciplinary team of experts in surgical quality measurement and the management of thyroid cancer, including endocrinologists, endocrine surgeons, and general surgeons. Through a series of consensus discussions, the team critically evaluated each candidate variable, considering factors such as its reliability, validity, impact on patient outcomes, alignment with current guidelines, and ease of data collection. Potential indicators were ranked based on their importance and feasibility. In January 2023, the MSQC started a collection of these new variables across all participating hospitals after training all nurse abstractors.

### 2.3 Statistical analysis

For this study, we analyzed the first year of data collection for these new variables (January–December 2023). Patients with a postoperative diagnosis of thyroid cancer (ICD C73) were included in the analysis. Descriptive statistics were used to characterize the study population and each potential quality indicator. To assess the quality of thyroid cancer surgical care, the performance on each quality indicator was evaluated and compared to current national guidelines for management of thyroid cancer.^12^, ^21^ The extent of thyroidectomy was determined by the chart abstraction of a single operative CPT code and reported as follows: thyroid lobectomy (60,220), total thyroidectomy (60,240), completion thyroidectomy (60,260), total thyroidectomy with a central neck dissection (CND; 60,252), and total thyroidectomy with a modified radical neck dissection (MRND; 60,254). All analyses were performed using Stata/SE version 18 (StataCorp, College Station, TX). This study was exempt from regulation by the University of Michigan Institutional Review Board, as it involved secondary analysis of de‐identified data collected for QI purposes.

## 3 RESULTS

### 3.1 Quality indicators selection

After multidisciplinary consensus discussion, the team of thyroid cancer and surgical quality experts selected 10 key quality indicators specific to thyroid cancer surgical care (Table 1). The selected preoperative quality indicator was the performance of FNA with cytologic Bethesda classification. Several quality indicators were chosen to assess the extent and quality of surgery including postoperative surgical pathology to determine cancer size, margin status, extrathyroidal extension, and lymph node status. Postoperative quality indicators included the occurrence of thyroidectomy‐specific complications during hospitalization and after hospitalization within 30 days of discharge. The presence of a surgeon‐documented postoperative follow‐up treatment plan within 30 days and a surgeon‐documented surveillance plan were also selected (Table 1). Because these variables are surgeon‐documented, they capture follow‐up treatment and surveillance plans regardless of referral and follow‐up at outside institutions. The number of variables was limited to 10 to balance the need for comprehensive data collection with the practical constraints of data abstraction and the burden on participating hospitals.

**TABLE 1:** Ten thyroid cancer‐specific quality indicators spanning the continuum of thyroid cancer surgical care, from preoperative evaluation to postoperative follow‐up, selected and included in the Michigan Surgical Quality Collaborative.

Variable	Definition	Aspect of care targeted
Preoperative thyroid nodule cytology	Preoperative Bethesda classification of cytology obtained by fine needle aspiration (FNA)	Preoperative evaluation
Postoperative surgical pathology	Examination and diagnosis of thyroid tissue removed during surgery	Extent and quality of surgery
Pathologic (TNM) staging	Determination of the extent of the primary tumor (T), involvement of regional lymph nodes (N), and presence of distant metastasis (*M*) of thyroid cancer based on surgical pathology	Extent and quality of surgery
Margin positivity	Presence or absence of thyroid cancer cells at the edge of the surgically removed thyroid tissue determined by surgical pathological examination	Extent and quality of surgery
Extrathyroidal extension	Extension of thyroid cancer beyond the thyroid gland, into adjacent tissues or structures, determined by surgical pathological examination	Extent and quality of surgery
Lymph node status	Presence or absence of thyroid cancer in the resected lymph nodes determined by surgical pathological examination	Extent and quality of surgery
Immediate postoperative complications	Adverse events or complications that occur during the patient's postoperative hospitalization, includes bleeding requiring reopening of incision, postoperative nausea and vomiting, recurrent laryngeal nerve injury, and hypocalcemia requiring calcium supplementation	Postoperative outcome
30 day postoperative complications	Adverse events or complications that occur within 30 days of hospital discharge, includes bleeding or symptomatic hematoma, recurrent laryngeal nerve injury, hypocalcemia requiring treatment, and airway symptoms	Postoperative outcome
Follow‐up treatment plan	Surgeon‐documented postoperative plan for additional treatment, includes no additional treatment, radioactive iodine therapy, and/or thyroid hormone supplementation	Postoperative outcome
Cancer surveillance plan	Surgeon‐documented postoperative plan for monitoring thyroid cancer recurrence or progression after surgery, includes clinical examinations by oncology/endocrinology/surgeon, ultrasound surveillance, calcitonin monitoring, and/or thyroglobulin monitoring	Postoperative outcome

### 3.2 Demographics

From January through December 2023, 360 patients with a postoperative diagnosis of thyroid cancer underwent thyroidectomy at 51 hospitals by 112 surgeons (Table 2). The MSQC sampling strategy captured a median of two cases per surgeon (IQR 1–4 cases; min 1, max 16). The mean patient age was 51.5 ± 16.1 years. Patients were 75.0% (*n* = 270) female and 80.3% (*n* = 289) White. Overall, 42.3% (*n* = 146) underwent total thyroidectomy and 28.4% (*n* = 98) received thyroid lobectomy. In addition to those undergoing total thyroidectomy alone, 19.4% (*n* = 68) underwent total thyroidectomy with a CND and 2.9% (*n* = 10) with a MRND. 6.6% (*n* = 23) of patients underwent completion thyroidectomy. Postoperative pathology demonstrated 28.9% (*n* = 86) of cancers measured <1 cm, 52.2% (*n* = 188) were 1–4 cm, and 13.3% (*n* = 48) were >4 cm. Preoperative FNA results, final surgical pathology, and TNM staging are shown in Table 2.

**TABLE 2:** Demographic and clinical characteristics of patients undergoing thyroidectomy for thyroid cancer from January–December 2023.

Characteristic		Total—*n* (%)
Age—mean (SD)		51.5 (16.1)
Sex	Female	270 (75)
	Male	90 (25)
Race	White	289 (80.2)
	Black or African American	26 (7.2)
	Other/multiracial^*^	17 (4.7)
	Unknown	28 (7.8)
Ethnicity	Hispanic	12 (3.3)
	Non‐Hispanic	321 (89.2)
	Unknown	27 (7.5)
Preoperative FNA	Bethesda I (nondiagnostic)	4 (1.1)
	Bethesda II (benign)	15 (4.2)
	Bethesda III (AUS/FLUS)	55 (15.3)
	Bethesda IV (follicular neoplasm)	22 (6.1)
	Bethesda V (suspicious for malignancy)	32 (8.9)
	Bethesda VI (malignant)	75 (20.8)
	Not performed	109 (30.3)
	Not documented/Missing	48 (13.3)
Extent of thyroidectomy	Total thyroidectomy	146 (42.3)
	Thyroid lobectomy	98 (28.4)
	Total thyroidectomy with CND	68 (19.7)
	Total thyroidectomy with MRND	10 (2.9)
	Completion thyroidectomy	23 (6.7)
Surgical pathology result	Papillary carcinoma	283 (78.6)
	Follicular carcinoma	29 (8.1)
	Poorly differentiated carcinoma	6 (1.7)
	Medullary carcinoma	3 (0.8)
	Anaplastic carcinoma	1 (0.3)
	Benign tumor/Nodule	11 (3.1)
	Not documented/Missing	28 (7.8)
Tumor size	<1 cm	86 (23.9)
	1–4 cm	188 (52.2)
	>4 cm	48 (13.3)
Staging (TNM)	Stage I	271 (75.3)
	Stage II	39 (10.8)
	Stage III	2 (0.6)
	Stage IVa/IVb	5 (1.4)

*Note*: Abbreviations: AUS, Atypic of undetermined significance; FLUS, follicular lesion of undetermined significance; FNA, Fine needle aspiration; CND, Central neck dissection; RND, Radical neck dissection; SD, Standard deviation.

^*^ Includes American Indian or Alaska Native, Native Hawaiian or Pacific Islander, and Asian.

### 3.3 Preoperative evaluation

Preoperative evaluation included the completion of FNA with reported cytology results (Table 2). FNA was not performed in 34.3% (*n* = 103). Of those nodules without preoperative FNA results (*n* = 103), 30.1% (*n* = 31/103) measured <1 cm, 49.5% (*n* = 51/103) were 1–4 cm, and 20.4% (*n* = 21/103) were >4 cm. For cancers measuring <1 cm (*n* = 79), 48 (60.8%) nodules underwent preoperative FNA biopsy, and 16 (20.3%) were diagnosed as Bethesda V or VI, which may represent overdiagnosis per current clinical practice guidelines which recommend against the biopsy of nodules smaller than 1 cm.

### 3.4 Extent and quality of surgery

**FIGURE 1:**
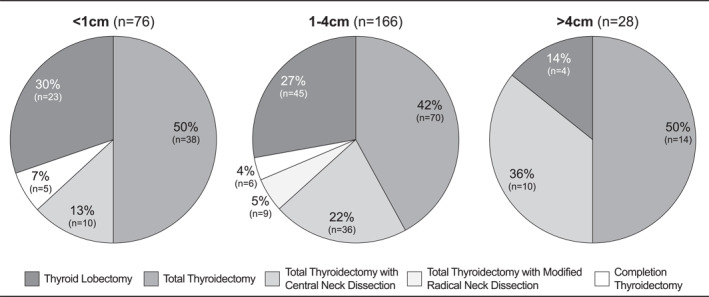
Extent of thyroidectomy varied by papillary thyroid cancer size with potential overtreatment of small cancers <1 cm with total thyroidectomy and undertreatment of larger cancers >4 cm with thyroid lobectomy based on current clinical practice guidelines.

When the extent of thyroidectomy was evaluated by cancer size on final surgical pathology, 46.9% (*n* = 38) of patients with <1 cm papillary thyroid cancers underwent total thyroidectomy, while 13.8% (*n* = 4) of patients with papillary cancers >4 cm underwent thyroid lobectomy (Figure 1). These findings suggest the potential overtreatment of small papillary cancers <1 cm that do not meet guideline recommendations for thyroidectomy and undertreatment of larger cancers >4 cm for which total thyroidectomy is indicated by current clinical practice guidelines.

Positive margins were found in 16.2% (*n* = 53) of patients on final pathology report. Of those with positive margins, 19.6% (*n* = 10/53) had extrathyroidal extension (ETE) on final pathology. Of all patients, 8.1% (*n* = 26) had ETE.

**FIGURE 2:**
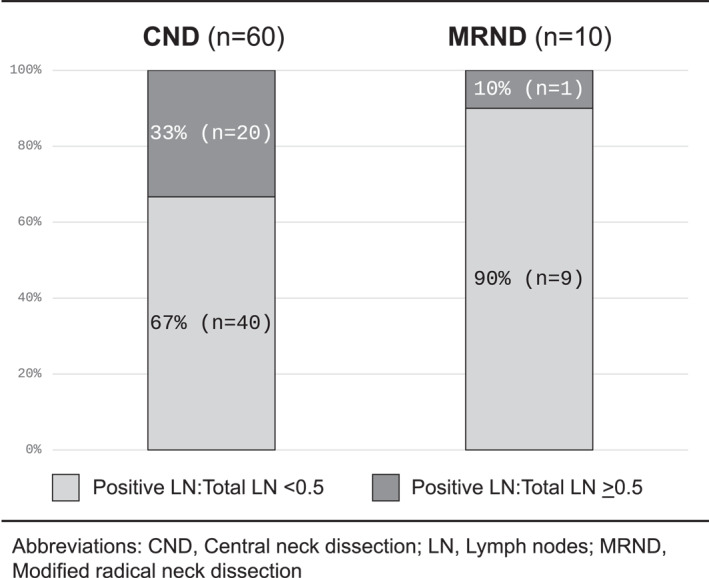
Lymph node ratios, defined as the number of positive nodes compared to the total number of lymph nodes removed, were ≥0.5 in 33% of central neck dissections and 10% of modified radical neck dissections performed.

Evaluation of lymph node status found that the median lymph node yield for CND was 11 (IQR 3–22.5) nodes versus 28 (IQR 9–35) nodes for MRND. However, only the index thyroidectomy CPT code was available for analysis, so some patients who underwent CND may have also had a bilateral procedure or MRND. Zero lymph nodes were found on final pathology in 11.7% (*n* = 7) of CND and 0% (*n* = 0) of MRND. When evaluating lymph node ratios, defined as the number of positive nodes compared to the total number of lymph nodes removed, 33.3% (*n* = 20) of CND and 10% (*n* = 1) of MRND had lymph nodes ratios ≥0.5, suggesting potentially inadequate nodal dissection or advanced burden of nodal disease (Figure 2). When evaluating CND by papillary cancer size, 17.9% (*n* = 10/56) were performed in patients where the primary cancer measured <1 cm, 64.3% (*n* = 36/56) were in patients with cancers 1–4 cm, and 17.9% (*n* = 10/56) were in patients with >4 cm cancers.

### 3.5 Postoperative outcomes

Evaluation of postoperative outcome variables included immediate complications during initial hospitalization and complications occurring within 30 days of surgery as well as surgeon‐documented postoperative follow‐up treatment and surveillance plans (Table 3). Evaluation of complications demonstrated that 10.6% (*n* = 38) of patients experienced complications immediately postoperatively during hospitalization, while 5.3% (*n* = 19) experienced complications after discharge within 30 days of surgery. Of those patients (*n* = 38) with immediate postoperative complications, 8.6% (*n* = 31/360) experienced hypocalcemia requiring calcium supplementation, 1.1% (*n* = 4/360) had a recurrent laryngeal nerve injury, 1.1% (*n* = 4/360) had postoperative nausea and vomiting, and 0.3% (*n* = 1/360) required reoperation for a neck hematoma (Table 3).

**TABLE 3:** Postoperative outcomes of patients undergoing thyroidectomy for thyroid cancer from January–December 2023.

Variable		Total—*n* (%)
Immediate postoperative complication	Bleeding requiring the reopening of incision	1 (0.2)
	Postoperative nausea and vomiting	4 (1.1)
	Recurrent laryngeal nerve injury	4 (1.1)
	Hypocalcemia requiring calcium supplementation	31 (8.6)
Post‐discharge, within 30 day postoperative complication	Bleeding or symptomatic hematoma	0 (0.0)
	Recurrent laryngeal nerve injury	4 (1.1)
	Hypocalcemia requiring calcium supplementation	15 (4.2)
Surgeon‐documented follow‐up treatment plan	Thyroid hormone supplementation	193 (53.6)
	Radioactive iodine therapy	48 (13.3)
	No additional treatment	77 (21.4)
	Plan not documented by surgeon	69 (19.2)
Surgeon‐documented cancer surveillance plan	Clinical exam by oncology/endocrinology/surgery	285 (79.2)
	Ultrasound surveillance	54 (15.0)
	Calcitonin monitoring	27 (7.5)
	Thyroglobulin monitoring	34 (9.4)
	Plan not documented by surgeon	67 (18.6)

When evaluating postoperative follow‐up and surveillance plans documented by their surgeon, 19.2% (*n* = 69) of patients lacked surgeon‐documented follow‐up treatment plans within 30 days postoperatively. Additionally, 18.6% (*n* = 67) lacked surgeon‐documented thyroid cancer surveillance plans (Table 3).

## 4 DISCUSSION

This study reports the successful development of a thyroid cancer‐specific QI program within an existing statewide SQC through the integration of 10 key quality indicators spanning the continuum of thyroid cancer surgical care from preoperative evaluation to postoperative follow‐up. This achievement highlights the potential for adapting existing QI infrastructures to address the unique needs of patients with thyroid cancer. The first year of data collection revealed multiple areas of potential guideline‐discordant care and opportunities for QI initiatives as well as the need to add and adjust the set of variables.

One notable finding is the potential underutilization of preoperative FNA, which is standard of care for evaluating most thyroid nodules prior to surgery.^12^ Our data showed that over a quarter of patients with >1 cm thyroid cancer on final pathology did not undergo preoperative FNA. Underutilization of FNA may lead to unnecessary diagnostic surgeries or inappropriate extent of index thyroidectomy. To help determine whether FNA is indicated in these cases, we have added an additional variable to the QI program in January 2024 to identify the preoperative indication for surgery. Surgeons may intentionally forgo an FNA in select patients with a multinodular or substernal goiter or Graves' disease. However, the initial variables did not allow us to assess this, and we plan to address this limitation in our updated data abstraction plan. We anticipate targeted interventions will be needed to improve adherence to preoperative evaluation guidelines such as audit and feedback or access to tele‐cytology. Audit and feedback have been shown to be effective in improving surgical quality in other malignancies.^5^, ^22^

Another area for potential QI initiatives is the extent of surgery performed based on papillary thyroid cancer size on final pathology. Over 10% of cancers measuring >4 cm were potentially undertreated with thyroid lobectomy. Current clinical practice guidelines recommend total thyroidectomy for cancers >4 cm given the increased risk of local recurrence and metastatic spread and potential need for radioactive iodine.^12^, ^21^ When examining cancers measuring <1 cm, we found that nearly half of these patients underwent total thyroidectomy. While some of these cases may represent incidental microcarcinomas found on final pathology for those undergoing total thyroidectomy for other reasons such as autoimmune disease or multinodular goiter, the high rate of total thyroidectomy for these small cancers raises concerns about potential overuse.^23^ Given these findings of potentially guideline‐discordant care, a strategy for QI is a strategic education‐based initiative that promotes appropriate thyroid nodule work‐up and management.

Overall, this study demonstrates the feasibility of collecting thyroid cancer‐specific surgical quality data and using it to identify areas for QI. Our thyroid‐specific QI initiative offers several advantages over existing national programs including nurse‐abstracted data and capture of a diverse range of practice settings. This is particularly important for thyroid cancer where many thyroidectomies are performed by low‐volume surgeons and at nonacademic medical centers. The successful implementation of the program within the MSQC highlights the potential for other regional or national collaboratives to adopt similar approaches to measuring and improving the quality of thyroid cancer surgical care. The next step will be using MSQC's established processes of data sharing, collaborative meetings, and sharing of best practices. We will also refine and potentially expand the quality indicators collected. Because MSQC presently collects 30 day outcomes, the implementation of other potential quality indicators that provide a more comprehensive assessment of thyroid cancer surgical quality, such as postoperative thyroglobulin levels at 3 months and 1 year and long‐term follow‐up data on recurrence and survival, may be challenging.^18^, ^19^, ^20^

This study has several limitations to consider. First, cases for review are selected based on postoperative diagnosis of thyroid cancer which limits the ability to completely assess surgical decision‐making. This limitation has been addressed; moving forward, the MSQC will collect preoperative surgical indication as an additional variable. Second, given data abstraction processes, we are unable to definitively determine if the preoperatively biopsied nodule was the cancer reported in the final pathology. Additionally, abstraction currently only captures tumor size data on the largest cancer if more than one focus was found on final pathology. Third, the relatively short duration of data collection (1 year) may not fully capture longer‐term trends or outcomes. Ongoing data collection and validation efforts will be necessary to further characterize patterns of care and make robust comparisons over time and between organizations. Lastly, these data were collected within the Michigan SQC, which may limit the generalizability to other states or countries. Nonetheless, the collaborative is made up of a diverse group of health care settings. Finally, the accuracy of the findings may be affected by inaccurate coding, potential data abstraction errors, poor clinical documentation leading to missing data, or inconsistencies in data entry, which are inherent challenges in any large‐scale QI initiative. Some of these limitations may be mitigated by selective chart audits and ongoing training of MSQC nurse abstractors.

## 5 CONCLUSION

The development of this thyroid cancer‐specific QI program highlights the potential for measuring and improving the quality of thyroid cancer surgical care within an existing SQC. Results of the first year of data collection have identified several targetable opportunities for QI initiatives including appropriateness of preoperative FNA and extent of thyroidectomy performed by cancer size. In addition to informing new QI initiatives, the results have led to improvement in thyroid cancer‐related data abstraction and QI processes within the MSQC. This novel program represents a reproducible opportunity to address gaps in thyroid cancer surgical QI and serves as a model that can be adapted by other institutions and collaboratives.

## AUTHOR CONTRIBUTIONS

**Catherine B Jensen**: Conceptualization; Formal analysis; Investigation; Methodology; Visualization; Writing–original draft; Writing ‐ review and editing. **Elizabeth M Bacon**: Project administration; Writing ‐ review and editing. **Lauren N Krumeich**: Writing–review and editing. **Hunter J Underwood**: Writing–review and editing. **David T Hughes**: Writing ‐ review and editing. **Paul G Gauger**: Writing–review and editing. **Richard Burney**: Conceptualization; Data curation; Methodology; Writing–review and editing. **Susan Pitt**: Conceptualization; Methodology; Resources; Supervision; Visualization; Writing–review and editing.

## CONFLICT OF INTEREST STATEMENT

The authors declare no conflicts of interest.

## ETHICS STATEMENT

This study was exempt from regulation by the University of Michigan Institutional Review Board, as it involved secondary analysis of de‐identified data collected for QI purposes.

## Data Availability

The data that support the findings of this study are available from the Michigan Surgical Quality Collaborative (MSQC). Restrictions apply to the availability of these data, which were used under license for this study. Data are available from the authors with the permission of MSQC.
